# Efficacy of Meal Replacement Products on Weight and Glycolipid Metabolism Management: A 90-Day Randomized Controlled Trial in Adults with Obesity

**DOI:** 10.3390/nu16193284

**Published:** 2024-09-28

**Authors:** Botian Chen, Shiyi Hong, Yuyang Wang, Qiang Hu, Defu Ma

**Affiliations:** School of Public Health, Peking University Health Science Center, Beijing 100191, China; cbt970812@163.com (B.C.); syhongh@163.com (S.H.); wyuyang2018@163.com (Y.W.); qianghu@bjmu.edu.cn (Q.H.)

**Keywords:** diet, meal replacement, adults with obesity, weight loss

## Abstract

**Background:** The global obesity issue is growing increasingly serious, impacting personal health, economic development, and the sustainability of medical systems. There is an urgent need for effective weight loss strategies that can be widely implemented. This study conducted a 90-day randomized controlled trial to assess the impact of meal replacement products on weight management and glycolipid metabolism in adults with obesity. **Methods:** Adults with obesity meeting the inclusion and exclusion criteria were divided into three groups: the meal replacement group (*n* = 19), the diet control group (*n* = 19), and the normal diet group (*n* = 22). The meal replacement group used specially formulated meal replacement products for dinner, and the diet control group reduced the intake of staple food at lunch, both controlling daily energy intake between 1200 kcal and 1300 kcal, while the normal diet group maintained their regular dietary habits. Relevant indicators were measured at baseline and after 45 and 90 days of intervention. **Results:** The results showed that both the meal replacement group and the diet control group experienced significant decreases in weight, BMI, and body fat percentage, with the meal replacement group showing a more pronounced weight loss effect. The weight loss of the meal replacement group at 45 and 90 days was 4.44 ± 1.84 kg and 7.38 ± 3.24 kg, the diet control group was 2.62 ± 2.28 kg and 4.08 ± 2.94 kg, and the normal diet group was 0.66 ± 1.73 kg and 0.97 ± 2.02 kg. The decrease in BMI at 45 and 90 days for the meal replacement group was 1.08 ± 0.78 kg/m^2^ and 2.17 ± 1.57 kg/m^2^, for the diet control group was 0.82 ± 0.80 kg/m^2^ and 1.39 ± 1.16 kg/m^2^, and for the normal diet group was 0.19 ± 0.71 kg/m^2^ and 0.21 ± 0.96 kg/m^2^. The decrease in body fat percentage at 45 and 90 days for the meal replacement group was 1.76 ± 0.68% and 3.67 ± 2.62%, for the diet control group was 1.02 ± 1.11% and 1.52 ± 1.79%, and for the normal diet group was 0.81 ± 1.09% and 0.53 ± 0.93%. In addition, the decrease in BMI and body fat percentage in the meal replacement group was also significantly higher than in the other two groups. In terms of metabolic indicators, there were no significant differences in the changes of blood pressure, blood lipids, blood sugar, and ALT levels among the three groups during the intervention period. **Conclusions:** In summary, the results indicate that meal replacement products can significantly reduce weight and body fat percentage without affecting metabolic health.

## 1. Background

Obesity has emerged as a pervasive global health concern, exerting a profound impact on personal well-being, economic progress, and the sustainability of healthcare systems. A stark increase in obesity rates has been documented over the last four decades, with the prevalence increasing from 3% to 11% among men and nearly tripling from 6% to 15% among women [1]. Excess weight has been consistently linked to a spectrum of chronic conditions, such as hypertension, diabetes, hyperlipidemia, and coronary heart disease [2,3,4]. Projections from the Global Obesity Map suggest that by 2035, the financial strain attributable to overweight and obesity will correspond to 2.9% of the global GDP [5]. China, as a developing nation with a relatively low per capita income, is witnessing a concerning upward trend in overweight and obesity rates across all age groups, with over 50% of adults and nearly 20% of children and adolescents affected [6]. Consequently, the scientific community has a keen interest in effective obesity management strategies.

A wealth of evidence supports the efficacy of exercise [7], dietary regulation [8], reduced sedentism [9], and other healthy behaviors in alleviating overweight and obesity to varying extents. However, certain misguided approaches to exercise and dietary management have been associated with adverse metabolic outcomes and functional impairments [10,11,12]. The execution of these weight reduction strategies often demands considerable time and financial investment [13]. Professional guidance from dietitians for dietary adjustments and trainers for exercise regimens is ideal but may be cost-prohibitive, limiting widespread adoption [14], particularly in less affluent regions. Against this backdrop, identifying an effective and scalable weight reduction approach is of paramount importance.

A potential micronutrient deficiency is a common concern with dietary control for weight loss, as restrictive eating patterns can limit the intake of essential vitamins and minerals. This concern underscores the importance of a well-rounded approach to weight management that ensures nutritional adequacy [15,16,17]. Research indicates that a sustained negative energy balance is essential for weight loss in individuals with overweight and obesity, and energy-restricted meal replacements may offer a viable and safe strategy for weight management [18]. Formulated to meet the nutritional requirements for one or two meals during weight control, meal replacements are specially processed energy-controlled food products [19], such as shakes, energy bars, powders, and pre-packaged meals [20]. Evidence suggests that these products can significantly and safely ameliorate obesity in adults [21]. Their affordability and ease of use position meal replacements as a potentially effective strategy for broad-scale weight management initiatives, although some studies have reservations about the appropriateness of using meal replacements for weight loss, primarily due to safety considerations regarding certain very low-calorie diet products [22,23]. The current research has largely employed meal replacements as a tool for dietary control (in comparison to non-intervention control groups) [18,24]. There is a scarcity of studies that delve into the effects of more nutritionally comprehensive meal replacements on weight loss and metabolic indicators in obese individuals, as compared to traditional dietary interventions.

The purpose of this study is to explore the real-world efficacy of meal replacement products in managing the weight and glycolipid metabolism of individuals who are overweight and obese, and to assess their advantages and disadvantages relative to dietary control. The main objective is to evaluate the impact of meal replacement products on weight reduction in this population and to compare their effects on metabolic profiles with those of traditional dietary control methods. This will shed light on the strengths and limitations of meal replacements as a weight loss intervention, providing real-world evidence for a more straightforward and broadly applicable weight reduction strategy.

## 2. Materials and Methods

### 2.1. Ethics Statement

The study protocol was approved by the Ethics Committee of Peking University Health Science Center on 21 October 2022 (NO. IRB00001052-22128).

### 2.2. Study Participants

We distributed recruitment advertisements through a health management company, targeting potential research participants in the communities of Beijing and its surrounding areas. All potential participants were required to read and sign an informed consent form.

The specific inclusion criteria were as follows: 1. Males or females aged 18–65 years with a BMI ≥ 25.0 kg/m^2^ [5]; 2. ① Normal total cholesterol levels (TC < 6.2 mmol/L), ② Normal triglycerides levels (TG < 2.3 mmol/L) [25], ③ Normal blood pressure range (SBP < 140 mmHg, DBP < 90 mmHg) [26], ④ Normal fasting blood glucose levels (FBG < 7 mmol/L) [27], ⑤ Normal ALT Levels (ALT < 40 U/L) [28]; 3. No severe liver, kidney, or gastrointestinal diseases, or diabetes complications; 4. No use of lipid-lowering, blood sugar-lowering, or weight-related drugs and dietary supplements within 3 months, and agreement not to use the aforementioned substances during the experiment; 5. Voluntary participation in the study after hearing the project introduction and signing the informed consent form. Exclusion criteria included: 1. Women preparing for pregnancy, pregnant, or lactating; 2. Excessive smoking or alcohol consumption; 3. Special dietary habits: vegetarians, ketogenic dieters (high fat, low carbohydrate), etc.; 4. Allergies to soy or milk.

The sample size calculation was based on Huseinovic 2016, AJCN (Effectiveness of a weight loss intervention in postpartum women: results from a randomized controlled trial in primary healthcare) [29], with a dietary intervention group showing a weight loss of 6.1 ± 3.5 kg over 12 weeks, and a control group showing a weight loss of 2.4 ± 3.5 kg. With α = 0.05, β = 0.2, the calculated *n* was 15, and accounting for a 30% dropout rate, 22 participants were enrolled in each group.

**Figure 1:**
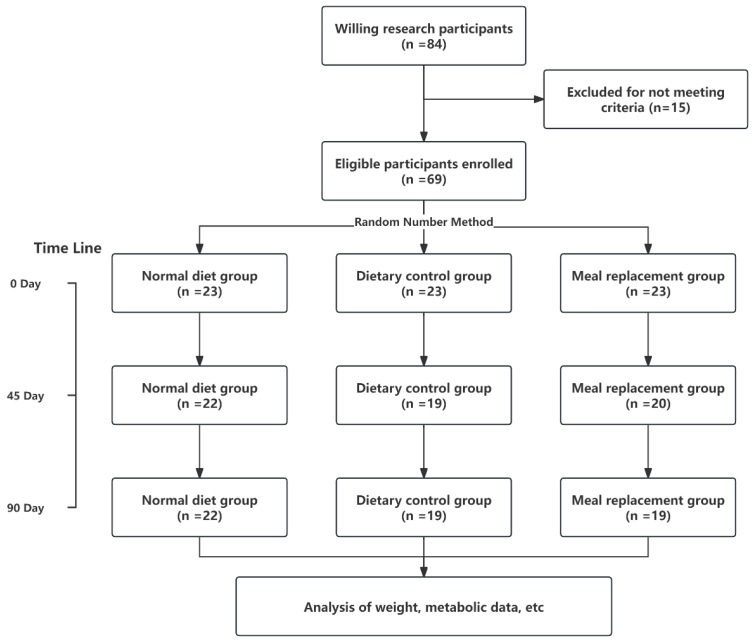
Study flow chart.

In the study’s recruitment phase, 84 volunteers were enrolled, with 66 qualifying based on the predefined eligibility criteria. Throughout the course of the intervention, six participants regrettably withdrew or were lost to follow-up (two individuals discontinued their involvement because they could not adhere to the dietary protocol, and four others had to withdraw for various personal reasons that made further study participation impossible), resulting in a final cohort of 60 dedicated subjects who completed the study. The distribution of these subjects was as follows: 22 in the NDG, 19 in the DCG, and 19 in the MRG.A visual representation of the study’s procedural flow is depicted in Figure 1, offering a clear overview of participant progression and retention.

### 2.3. Study Design

This study is a randomized controlled trial that divided eligible participants into three groups using a random number method: the meal replacement group, the diet control group, and the normal diet group, with 22 participants in each group. The meal replacement group (MRG) was intervened with a specially formulated meal replacement product (provided free of charge by Life Talk (Beijing) Health Management Co., Ltd. (Beijing, China).; each 100 g of the meal replacement contained 370 kcal of energy, 45 g of protein, 7.5 g of fat, 21.4 g of carbohydrates, and 571 mg of sodium; see Table S1 for specific ingredients), replacing dinner. Participants consumed two bags of the meal replacement product for dinner (each bag was 30 g, mixed with 400 mL of 50 °C water, stirred, and then consumed), and the daily energy intake of the participants in the meal replacement group was assessed, restricting the daily energy intake to a range of 1200–1300 kcal by controlling the intake of staple foods at lunch. The diet control group (DCG) controlled the amount of staple food intake to keep the daily energy intake within the same range, ensuring consistency with the MRG. To control the daily energy intake to a range of 1200–1300 kcal, participants in both the MRG and DCG were advised through dietary prescriptions to maintain their energy intake around this range. They were also asked to document their daily meals through food photography as a form of daily check-in, although this was not quantitatively assessed. Subsequently, nutritionists made approximate adjustments to the amount of staple food intake for each participant. The normal diet group (NDG) maintained their regular diet without additional intervention. The meal replacement products were distributed regularly (one week’s supply was provided to the participants each week), and the samples were stored in a dry, light-free environment at room temperature of 25 °C. A dedicated person (custodian) was responsible for recording the storage conditions and the sample’s entry and exit records. Each group of 11 participants was supported by a follow-up observer, who collected the trial samples from the custodian once a week, filled in the sample collection record, including the quantity, batch number, time, and signature, and distributed them to the participants, who then signed to confirm receipt.

### 2.4. Study Variables and Measurements

Baseline demographic information, such as gender and age, was collected at enrollment. Anthropometric measurements including height, weight, BMI (weight/height^2^), body fat percentage, systolic and diastolic blood pressure, fasting blood glucose, total cholesterol, triglycerides, and alanine aminotransferase were measured at baseline (day 0), and after 45 and 90 days of intervention. (The rationale for selecting ALT as a primary indicator includes: ① ALT’s Utility: ALT is a critical biomarker for assessing liver health and metabolism, essential for evaluating the safety and efficacy of our dietary interventions. ② Practical and Economic Considerations: ALT use streamlines our trial design, providing valuable health insights for participants while efficiently managing research resources [28]). Body fat percentage was assessed throughout the study using multifrequency bioelectrical impedance analysis with an eight-point tactile electrode system (ioi 353; Jawon Medical, Gyeongsan-si, Republic of Korea). Prior to the measurements, researchers entered the participants’ gender, age, and height data into the system. The bioelectric impedance measurements were taken within a 1–2 min window, with participants standing barefoot and holding the hand electrodes in a vertical arm position [30]. Weight was measured with the individual wearing light clothing and no shoes, using a calibrated electronic scale for accuracy. Height measurements were obtained using a standard wall-mounted stadiometer, recorded to the nearest 0.5 cm. An automatic sphygmomanometer was used to measure blood pressure in the left arm (positioned at heart level) while seated after at least 5 min of quiet rest. The measurement was taken at least 5 min apart, and the average of the two readings was recorded. Fasting venous blood samples were collected, and fasting blood glucose was measured using the glucose oxidase method per routine operating procedures. Total cholesterol, triglycerides, and other blood indicators were measured while fasting. All on-site measurements, including laboratory tests, were validated for internal quality control according to clinical standards.

### 2.5. Statistical Analysis

All statistical analyses were conducted using R statistical software, version 4.1.3. The baseline characteristics and clinical outcomes of the study participants were presented as means ± standard deviations for continuous variables and frequencies for categorical variables. Depending on the type of variable, Kruskal–Wallis tests (or one-way ANOVA if the data met the assumptions of normality and homogeneity of variance) and Pearson’s chi-square tests were used. Subsequently, Bonferroni’s *p* adjustment method or the Least Significant Difference (LSD) method was used for post-hoc pairwise comparisons, assuming statistical significance at *p* < 0.05.

## 3. Results

The study’s baseline characteristics revealed an NDG of 22 participants, equally divided by gender, alongside a DCG with 19 participants consisting of 10 males and nine females, and a MRG also with 19 individuals, including nine males and 10 females. At the outset, no statistically significant disparities were identified across the groups concerning demographic attributes, ensuring equitable group comparability. The mean ages for the NDG, DCG, and MRG were recorded as 41.73 ± 9.75 years, 43.16 ± 11.88 years, and 43.16 ± 10.38 years, respectively. Corresponding mean weights were 78.20 ± 16.51 kg, 80.47 ± 17.70 kg, and 83.20 ± 9.55 kg, with average BMIs of 29.10 ± 4.74 kg/m^2^, 30.16 ± 5.12 kg/m^2^, and 29.87 ± 3.24 kg/m^2^. Average body fat percentages were noted at 36.71 ± 6.55% for NDG, 36.24 ± 6.85% for DCG, and 35.93 ± 6.71% for MRG (Table 1).

### 3.1. Improvement in Anthropometric Measurements

**Figure 2:**
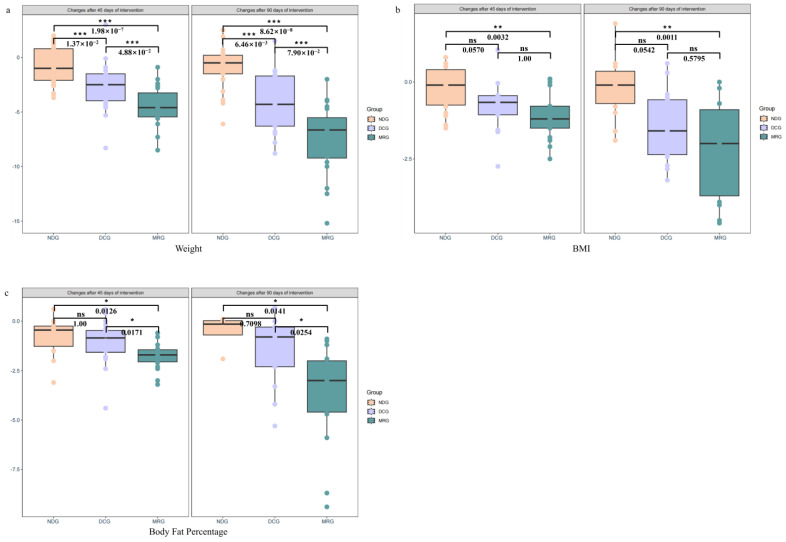
Changes in anthropometric measurements among the three groups of study subjects. Weight (**a**); BMI (**b**); Body Fat Percentage (**c**). NDG: Normal diet group; DCG: Diet control group; MRG: Meal replacement group. ***: *p* < 0.001; **: 0.001 < *p* < 0.01; *: 0.01 < *p* < 0.05; ns: no significant. Kruskal–Wallis test was used and pairwise test for multiple comparisons was performed using Bonferroni’s *p*-adjustment method.

Significant enhancements in anthropometric indices were observed in the MRG and DCG following the 45-day intervention, with respective weight losses of 4.44 ± 1.84 kg and 2.62 ± 2.28 kg, contrasting with a negligible 0.66 ± 1.73 kg decrease in the NDG. The MRG’s weight reduction was notably superior to both the DCG (*p* < 0.001) and NDG (*p* < 0.001), with the DCG also demonstrating a significant advantage over the NDG (*p* < 0.001). By day 90, the MRG and DCG groups exhibited even more pronounced weight losses of 7.38 ± 3.24 kg and 4.08 ± 2.94 kg, respectively, in stark contrast to the NDG’s minimal 0.97 ± 2.02 kg reduction. The MRG continued to outperform the DCG (*p* < 0.001) and NDG (*p* < 0.001), with the DCG maintaining a significant lead over the NDG (*p* < 0.001) (Figure 2a).

In terms of BMI, the MRG and DCG groups recorded reductions of 1.08 ± 0.78 kg/m^2^ and 0.82 ± 0.80 kg/m^2^ after 45 days, significantly exceeding the NDG’s modest 0.19 ± 0.71 kg/m^2^ decrease. The MRG’s BMI reduction was markedly higher than the NDG’s (*p* = 0.0032). At the 90-day juncture, the BMI reductions for the MRG and DCG were 2.17 ± 1.57 kg/m^2^ and 1.39 ± 1.16 kg/m^2^, respectively, while the NDG’s BMI only declined by 0.21 ± 0.96 kg/m^2^. The MRG’s BMI reduction remained significantly more substantial than the NDG’s (*p* = 0.0011) (Figure 2b).

Concerning body fat percentage, the MRG group’s 45-day intervention resulted in a 1.76 ± 0.68% decrease, significantly surpassing the DCG and NDG groups’ reductions of 1.02 ± 1.11% and 0.81 ± 1.09%, respectively (*p* = 0.0171 for MRG vs. DCG and *p* = 0.0126 for MRG vs. NDG). By the 90-day mark, the MRG group’s body fat percentage had significantly dropped by 3.67 ± 2.62%, in comparison to the DCG and NDG groups’ more modest decreases of 1.52 ± 1.79% and 0.53 ± 0.93%, respectively (*p* = 0.0254 for MRG vs. DCG and *p* = 0.0141 for MRG vs. NDG) (Figure 2c).

The study’s outcomes underscore the efficacy of meal replacement products in significantly ameliorating both body weight and body fat percentage, offering a discernible benefit over dietary control measures with equivalent energy intake, particularly in the realm of weight and body fat reduction.

### 3.2. Metabolic Parameters Status

**Figure 3:**
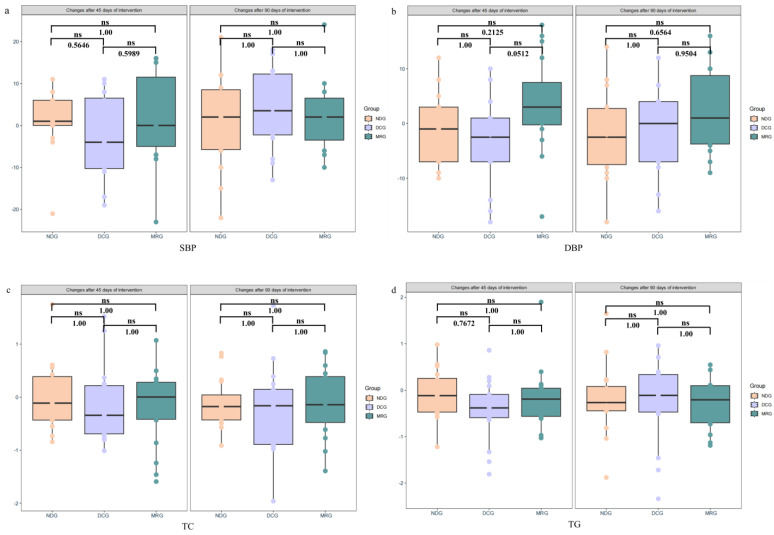

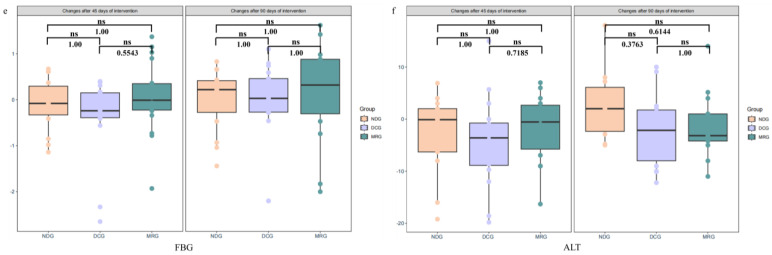
Changes in metabolic Indicators among the three groups of study subjects. SBP (**a**); DBP (**b**); TC (**c**); TG (**d**); FBG (**e**); ALT (**f**). NDG: Normal diet group; DCG: Diet control group; MRG: Meal replacement group. ns: no significant. Kruskal–Wallis test was used and pairwise test for multiple comparisons was performed using Bonferroni’s *p*-adjustment method.

Our analysis of metabolic parameters after both 45 and 90 days of intervention revealed no significant inter-group differences in blood pressure (Figure 3a,b), serum cholesterol (Figure 3c), triglycerides (Figure 3d), fasting blood glucose (Figure 3e), and liver enzyme ALT levels among the MRG, DCG, and NDG groups (Figure 3f). Notably, the MRG exhibited consistent safety with no indications of liver damage, as evidenced by stable ALT levels, supporting the notion that meal replacement products can be incorporated into weight reduction programs without adverse effects on liver health.

## 4. Discussion

The results of this study indicated that in comparison to the normal diet group, both the consumption of meal replacement products and the implementation of dietary control measures effectively reduced body weight and decreased body fat percentage, with the meal replacement group demonstrating superior efficacy in these reductions. Additionally, as the duration of the intervention was extended, the meal replacement group showed an increasingly pronounced divergence from the dietary control group, indicating a more favorable trend. 

The benchmark for effective weight reduction is generally considered to be a 5% to 10% decrease from the baseline body weight [31]. Prior research, potentially constrained by shorter intervention periods or variations in meal replacement compositions, reported a mere 4.2% weight loss after 12 weeks [19]. In contrast, our study achieved an 8.87% weight reduction in the meal replacement group following a 90-day intervention, signifying that meal replacements can indeed meet the target for effective weight loss. A weight loss of 5% is also widely recognized as having clinical significance [32]. Research by Knowler et al. found that a 5.95% weight decrease can reduce the risk of diabetes by 58% [33]. The Look AHEAD Research Group revealed that when individuals with obesity lose more than 10% of their body weight within a year, their risk of death from cardiovascular diseases is reduced by 21%, and their risk of developing cardiovascular diseases is lowered by 24% [34]. For individuals with obesity, weight loss can significantly mitigate the risk of various chronic diseases. Our findings suggest that meal replacement for weight loss is efficacious and that its scientific use may also mitigate the risks of diabetes and cardiovascular diseases among individuals with obesity to a certain extent. According to the World Health Organization (WHO), obesity is a chronic complex disease characterized by excessive fat deposits that can impair health [35], and body fat percentage reflects the amount of fat in the human body [36]. Our study observed a significant reduction in body fat percentage in the meal replacement group after a 90-day intervention, consistent with previous findings on meal replacements [37], indicating that a higher proportion of the weight loss from meal replacements is derived from fat, which also implies a relative increase in muscle mass, typically associated with healthier body composition and improved physical functionality [38].

While previous studies have not reached a consensus on whether weight loss can lead to a decrease in blood pressure, blood lipids, and blood sugar, some research indicates that weight reduction can indeed lower these metrics in individuals with obesity. For instance, Steger et al. noted significant improvements in blood pressure after a 6-month weight loss intervention [39], and Roebroek et al. observed a marked decrease in blood sugar levels in adolescents with obesity post-weight loss [40]. Headland et al. also found that sustained weight loss over time can effectively reduce blood pressure, blood lipids, and blood sugar levels [41]. Conversely, numerous studies have reported no changes in these metabolic indicators following weight loss in individuals with obesity. Aucott et al. concluded in their systematic review that weight loss did not improve hypertension [42], and Gepner et al. found no alteration in blood pressure after an 18-month randomized controlled trial [43]. A clinical study reported no significant changes in blood sugar and blood lipids post-weight loss [44]. Our study did not detect significant fluctuations in blood pressure, blood lipids, blood sugar, or ALT levels in either the meal replacement or dietary control groups, which might be related to the initial normal levels of these metrics at baseline and warrants further investigation. Moreover, Suzuki et al. found that rapid weight loss might cause liver damage, leading to elevated ALT levels in the blood [45]. However, our study found no significant changes in ALT levels in the meal replacement group, with a baseline weight loss of 8.87% and a body fat percentage decrease of 3.67%, suggesting that weight loss through meal replacement is safe to some extent.

Empirical evidence has established that dietary restriction and meal replacement strategies are both viable for weight reduction [46,47], a conclusion that finds resonance with our study’s outcomes. Importantly, our research suggests that the efficacy of meal replacement in weight loss is notably enhanced. A substantial body of research has highlighted the risk of micronutrient deficiencies associated with restrictive dietary weight loss approaches [48]. Conversely, meal replacements are expertly designed to deliver a comprehensive profile of essential vitamins and minerals, even with their low-calorie formulation. They specifically address the common nutritional shortfalls observed in populations with obesity [49], including zinc, folate, vitamins B1 and B12, A, E, and D [50,51,52], thereby offering a more balanced and healthful weight management solution. Furthermore, stringent dietary management demands a significant investment of time [53] and a high level of self-discipline and nutritional acumen, attributes that are challenging for the layperson to muster without expert counsel [54,55]. Meal replacement offers a fitting solution for individuals leading hectic lives or those not well-versed in nutrition. The predefined caloric structure of these replacements ensures accurate energy intake regulation, thereby markedly improving the adherence to a weight loss regimen [56,57].

In summary, we can infer that weight loss through meal replacement, while effective, does not lead to adverse changes in metabolic indicators such as blood pressure, blood lipids, blood sugar, and ALT levels.

This study does have some limitations. Firstly, the intervention period of 90 days, though sufficient to observe initial changes, may not be long enough to assess long-term effects and sustainability. Secondly, despite a calculated and reasonably allocated sample size based on previous research, the overall number of participants is relatively small, potentially limiting the generalizability and statistical power of the findings. An additional limitation is that the energy intake for the meal replacement and diet control groups was suggested via dietary prescriptions without quantitative assessment. However, to monitor compliance and dietary habits, participants were asked to take photos of their daily meals as a form of check-in. This method, while not providing exact nutritional data, offered a practical way to track dietary adherence and patterns. Additionally, we acknowledge not controlling for exercise variability, which aligns with real-world diversity and reflects the practical effectiveness of meal replacements without prescribed physical activity. Furthermore, factors such as emotional states, which can influence metabolic indicators, are challenging to confirm and collect in real-time during the study. Lastly, while there were differences in certain demographic characteristics among the three groups, it is important to note that all participants were recruited from communities surrounding Beijing, suggesting a relatively consistent socioeconomic status across the study population. This homogeneity in geographical and socioeconomic background may mitigate some of the variability that could be introduced by socio-economic disparities but also limits the diversity of the sample. Despite these limitations, the observed changes in body weight and body fat percentage are statistically significant and hold clinical relevance. Future studies with larger and more diverse populations, as well as longer intervention periods, will be necessary to further validate these findings and explore their broader applicability.

## 5. Conclusions

Through a 90-day randomized controlled trial, this investigation assessed the application efficacy of meal replacement products in managing body weight and glycolipid metabolism. The outcomes demonstrate that these products are potent instruments for weight management, achieving a notable decrease in both body weight and body fat ratio, all the while preserving metabolic well-being. This study lays down a scientific foundation for the creation and dissemination of cost-effective weight reduction tactics. Nonetheless, additional research is essential to thoroughly comprehend the enduring impacts and underlying mechanisms of meal replacements across various demographic groups.

## Figures and Tables

**Table 1:** Baseline characteristics of the three groups of study subjects.

	NDG (*n* = 22)	DCG (*n* = 19)	MRG (*n* = 19)	*p*-Value
Gender				0.9487
Male	11 (50.00%)	10 (52.63%)	9 (47.37%)	
Female	11 (50.00%)	9 (47.37%)	10 (52.63%)	
Age, year	41.73 (9.75)	43.16 (11.88)	43.16 (10.38)	0.8820
Weight, kg	78.20 (16.51)	80.47 (17.70)	83.20 (9.55)	0.5317
BMI, kg/m^2^	29.10 (4.74)	30.16 (5.12)	29.87 (3.24)	0.7257
Body fat percentage, %	36.71 (6.55)	36.24 (6.85)	35.93 (6.71)	0.9564
SBP, mmHg	123.90 (20.20)	123.26 (16.67)	120.71 (16.29)	0.8514
DBP, mmHg	77.43 (14.06)	79.47 (12.26)	73.53 (9.25)	0.3427
TC, mmol/L	5.11 (0.99)	5.12 (1.05)	4.51 (1.05)	0.1056
TG, mmol/L	2.01 (1.51)	1.59 (0.83)	1.47 (0.63)	0.2368
FBG, mmol/L	5.38 (0.70)	5.67 (1.54)	5.44 (1.08)	0.6868
ALT, U/L	31.57 (26.45)	28.53 (17.04)	31.86 (25.13)	0.9561

Data are *n* (%) or mean (SD). NDG: Normal diet group; DCG: Diet control group; MRG: Meal replacement group; BMI: Body Mass Index; SBP: Systolic Blood Pressure; DBP: Diastolic Blood Pressure; TC: Total Cholesterol; TG: Triglycerides; FBG: Fasting Blood Glucose; ALT: Alanine Aminotransferase.

## Data Availability

The data presented in this study are available on request from the corresponding author due to privacy concerns.

## Supplementary Materials

The following supporting information can be downloaded at: https://www.mdpi.com/article/10.3390/nu16193284/s1, Table S1: Main Nutritional Components of the Meal Replacement.
