# Supplementary material for: Efficacy of Meal Replacement Products on Weight and Glycolipid Metabolism Management: A 90-Day Randomized Controlled Trial in Adults with Obesity
Source: Nutrients. 2024 Sep 28;16(19):3284. doi: 10.3390/nu16193284 (PMC11479124; doi:10.3390/nu16193284)
Supplement: Supplementary file 1 [file nutrients-16-03284-s001.zip › Supplemental Table S1.pdf]

Table S1. Main Nutritional Components of the Meal Replacement

**Contents**	Per 100 g
Energy, kcal	370
Protein, g	45
Fat, from non-trans fatty acids, g	7.5
Carbohydrates, g	21.4
Dietary Fiber, g	17.8
Sodium, mg	571
Vitamin A, μg	250
Vitamin D, μg	5.8
Vitamin E	11.25mg Alpha-Tocopherol Equivalents
Vitamin B1, Thiamine, mg	2.75
Vitamin B2, Riboflavin, mg	1.33
Vitamin B6, mg	1.08
Vitamin B12, μg	2.75
Vitamin C, mg	70.6
Niacin, mg	13.83
Folic Acid, μg	180
Pantothenic Acid, mg	4.83
Potassium, mg	288
Magnesium, mg	227
Calcium, mg	598
Iron, mg	8.4
Zinc, mg	10
L-Carnitine, g	0.834
Taurine, g	0.375
